# The Beta-, Neutrino- and Proton-Asymmetry in Neutron β-Decay

**DOI:** 10.6028/jres.110.056

**Published:** 2005-08-01

**Authors:** H. Abele, S. Baeßler, M. Deissenroth, F. Glück, J. Krempel, M. Kreuz, B. Märkisch, D. Mund, M. Schumann, T. Soldner

**Affiliations:** Physikalisches Institut der Universität Heidelberg, 69120 Heidelberg, Germany; Physikalisches Institut der Universität Mainz, Staudingerweg 7, D-55128 Mainz, Germany; Physikalisches Institut der Universität Heidelberg, 69120 Heidelberg, Germany; Physikalisches Institut der Universität Mainz, Staudingerweg 7, D-55128 Mainz, Germany; Physikalisches Institut der Universität Heidelberg, 69120 Heidelberg, Germany; Physikalisches Institut der Universität Heidelberg, 69120 Heidelberg, Germany; Institut Laue-Langevin, B.P. 156, F-38042 Grenoble Cedex 9, France; Physikalisches Institut der Universität Heidelberg, 69120 Heidelberg, Germany; Institut Laue-Langevin, B.P. 156, F-38042 Grenoble Cedex 9, France

**Keywords:** angular-correlation coefficients, neutron *β*-decay

## Abstract

This article describes measurements of angular-correlation coefficients in the decay of free neutrons with the superconducting spectrometer PERKEO II. A method for measuring the *β*-asymmetry coefficient *A* is presented, as well as a new method for determining the neutrino-asymmetry coefficient *B*, which allows a value for the proton-asymmetry coefficient *C* to be obtained for the first time. An ongoing experiment is trying to improve the accuracy of these quantities.

## 1. Introduction

Two parameters describe neutron *β*-decay within the Standard Model. One parameter is the first entry *V_ud_* of the quark-mixing Cabibbo-Kobayashi-Maskawa (CKM)-matrix. The other one is *λ*, the ratio of the axial vector and vector coupling constants. Our knowledge on *λ* comes from the *β*-asymmetry coefficient *A*, the correlation between neutron spin and the electron momentum, and with less precision from the coefficient *a*, the correlation between neutrino and electron momenta. With *λ* and the neutron lifetime *τ*, we determine the first CKM element *V_ud_*. The neutrino-asymmetry coefficient *B*, the correlation between neutron spin and neutrino momentum is rather insensitive to *λ*, but it might point to physics beyond the Standard Model emerging from supersymmetry or other Grand Unified Theories (GUT). Coefficient *C*, the correlation between neutron spin and proton momentum, is also sensitive to *λ* and can be used for a determination of *V_ud_*.

This article describes ongoing measurements of the correlations *A, B*, and *C* with the instrument PERKEO II from Heidelberg University. It is installed at the Institute Laue-Langevin in Grenoble. Results of the coefficients *B* and *C* from a previous measurement are presented. *C* is a combination of *A* + *B* [1] and has been measured for the first time.

## 2. *β*-Asymmetry *A* and Quark Mixing

The coefficient *A* is linked to the probability that an electron is emitted with angle *ϑ* with respect to the neutron spin polarization *P*:

$$W(\vartheta )=1+\frac{v}{c}PA\cos(\vartheta ),$$ (2.1)

where *v/c* is the electron velocity expressed in fractions of the speed of light. Neglecting order 1 % corrections, *A* is a simple function of *λ*:

$$A=-2\frac{\lambda (\lambda +1)}{1+3\lambda^{2}},$$ (2.2)

where we have assumed that *λ* is real.

For a measurement of *A*, the instrument PERKEO II has been installed at the PF1B cold neutron beam position at the High Flux Reactor at the Institut Laue-Langevin, Grenoble. The neutrons are polarized by two (8 × 8) cm^2^ supermirror polarizers in crossed geometry. The main component of the PERKEO II spectrometer is a superconducting 1.1 T magnet in a split pair configuration, with a coil diameter of about one meter. Neutrons pass through the spectrometer, whereas decay electrons are guided by the magnetic field to either one of two scintillation detectors with photomultiplier readout. The detector’s solid angle of acceptance is truly 2 × 2π above a threshold of 40 keV. Electron backscattering effects, serious sources of systematic uncertainty in *β*-spectroscopy, are effectively suppressed.

The measured electron spectra $N_{i}^{↑}\;(E_{e})$ and $N_{i}^{↓}\;(E_{e})$ in the two detectors (*i* = 1,2) for neutron spin up and down, respectively, define the experimental asymmetry $A_{i_{\exp}}$ as a function of electron kinetic energy *E*_e_

$$A_{i_{\exp}}(E_{e})=\frac{N_{i}^{↑}\;(E_{e})-N_{i}^{↓}\;(E_{e})}{N_{i}^{↑}\;(E_{e})+N_{i}^{↓}\;(E_{e})}.$$ (2.3)

$A_{i_{\exp}}$ is directly related to the asymmetry parameter *A*. Earlier experiments of instrument PERKEO gave a value of *A*_0_ = −0.1189(7) and *λ* = −1.2739(19) [3] after a 2 % correction for small experimental systematic effects. Other experiments with larger corrections on *A* [4.6] gave significantly lower values for *λ*.

The Standard Model describes quark-mixing with the CKM-matrix. This matrix remains unexplained in this theory. With instrument PERKEO, |*V_ud_*| = 0.9717(13) was obtained. The main contribution to the overall ±0.0013 uncertainty is the experimental error from the *β*-asymmetry *A* with ±0.0012. With |*V_us_*| = 0.2196(23) and the negligibly small |*V_ub_*| = 0.0036(9), one obtains

$$|V_{ud}{|}^{2}+|V_{us}{|}^{2}+|V_{ub}{|}^{2}=1-\Delta =0.9917(28).$$ (2.4)

This value differs from the Standard Model prediction by ∆ = 0.0083(28), or 2.7 times the stated uncertainty. Currently, superallowed 0^+^ → 0^+^ nuclear *β*-decay provides a value of |*V_ud_*| = 0.9740(5) [7], signaling a deviation from the Unitarity condition by 2.2 *σ* standard deviations. If the deviation is due to errors in |*V_us_*|, its presently accepted value would have to shift by 7 *σ* in order to explain the neutron result. However, very recent results [8,9] hint that the last word on |*V_us_*| has not yet spoken. An independent test of CKM unitarity comes from W physics at LEP where W decay hadronic branching ratios can be used. Since decay into the top quark channel is forbidden by energy conservation, one would expect Σ|*V_ij_*|^2^ to be 2 with a three generation unitary CKM matrix. The experimental result is 2.039(27), consistent with Eq. (2.4) but with considerably lower accuracy [10].

The main source for corrections in the experiment PERKEO so far have been neutron beam polarization (1.1 %) background (0.5 %) and flipper efficiency (0.3 %) with a total correction of 2.04 % to coefficient *A*. In the ongoing experiments, we have further reduced all corrections. With such small corrections to the data, a possible deviation from the Standard Model, if confirmed, will be seen very pronounced in the uncorrected data. Until now, major improvements both in neutron flux and degree of neutron polarization have been made: First, the University of Heidelberg has built a new ballistic supermirror guide for the ILL [11] which gives an increase of a factor of 4 in the cold neutron flux. Second, a new arrangement of two supermirror polarizers allows to achieve an unprecedented degree of neutron polarization *P*. The neutron polarization and the spin flip efficiency was measured to be *P* = 99.75(10) % (preliminary) and *f* = 100.0 % with an uncertainty of less than 0.1 % (preliminary) [2] over the full cross section of the beam. Third, systematic limitations of polarization measurements have been investigated: The beam polarization can now be measured with a completely different method using an opaque ^3^He spin filter with an uncertainty of 0.1 % [12]. As a consequence, we are now in the lucky situation to improve on the main uncertainties in reducing the main correction of 1.1 % to less than 0.25 % with an uncertainty of 0.1 %.

## 3. Neutrino-Asymmetry B and A Search for Right Handed Currents

Parity is maximally violated in the weak interaction. However, we do not have a fundamental justification. It is particularly interesting that modern grand-unified theories support a left-right symmetrical universe right after the start of the big bang. Parity violation arises only due to a spontaneous symmetry breaking at some intermediate energy scale. Parity violation is not 100 % and right handed contributions in the weak interaction should be found. Measurements of the correlation coefficient *B*, the correlation between neutrino momentum and neutron spin, are sensitive to right handed current contributions in the weak interaction. However, we have no evidence for right handed currents so far.

**Fig. 1:**
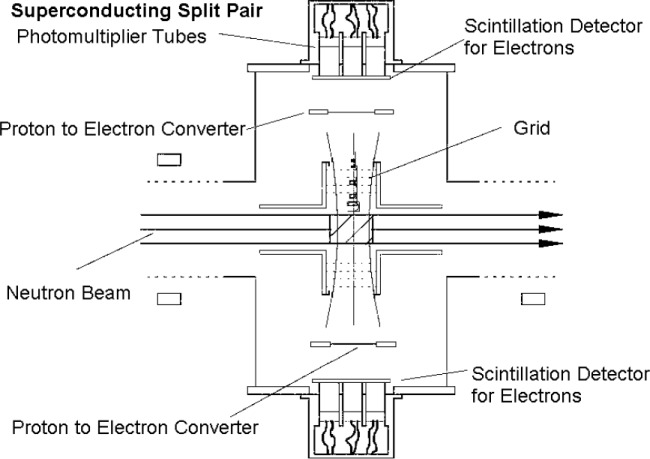
Setup for a measurement of coefficient *B* and *C*.

The spectrometer PERKEO II has been installed at the new beam position PF1B for a measurement of coefficient *B*. The basic principle of a coefficient *B* measurement is to measure the charged decay particles in neutron decay in order to reconstruct the neutrino momentum with respect to the neutron spin. Usually this is done with one electron and one proton detector. PERKEO uses a new method with one electron detector and one proton detector in each hemisphere. This is an advantage over other experiments because it maximizes the sensitivity on *B*. What is more, the measured asymmetry, which is proportional to *B*, shows only small electron energy dependence. Systematic uncertainties due to the detector response function are small. Fig. 1 shows the principle. The proton was measured in coincidence with a decay electron. The electron-detectors are made of plastic scintillators. The proton detectors also make use of the electron-detectors. The idea is to convert a proton into an electron signal. A proton will be accelerated up to 30 keV and eventually hit a thin foil of carbon. One proton creates about five secondary electrons being guided to the electron detectors.

In our setup with these combined electron-proton detectors on both hemispheres, we are able to define two observable asymmetries:

$$B_{i_{\exp,1}}(E_{e})=\frac{N_{i}^{↑↑↑}(E_{e})-N_{i}^{↓↑↑}(E_{e})}{N_{i}^{↑↑↑}(E_{e})+N_{i}^{↓↑↑}(E_{e})}\;\text{electron and proton detected in the same hemisphere}$$ (3.1)

$$B_{i_{\exp,2}}(E_{e})=\frac{N_{i}^{↑↑↓}(E_{e})-N_{i}^{↓↑↓}(E_{e})}{N_{i}^{↑↑↓}(E_{e})+N_{i}^{↓↑↓}(E_{e})}\;\text{electron and proton detected in the same hemisphere}$$ (3.2)

**Fig. 2:**
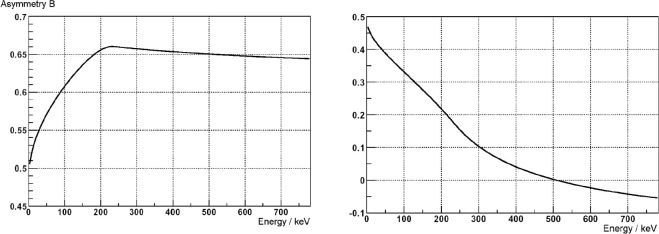
Expected electron-proton asymmetry in dependence of the electron energy for both possible configurations. Left: both particles detected in the same hemisphere. Right: both particles detected in opposite hemispheres.

The arrows indicate the direction of the neutron spin and the hemisphere direction of the electron and proton respectively. The resulting dependence of the asymmetry from the electron energy can be seen in Fig. 2. If the electron and the proton are detected in the same hemisphere, the asymmetry is rather insensitive to the electron energy and thus insensitive to the detector calibration and resolution. In addition, the influence of other asymmetry coefficients is suppressed and a high statistical sensitivity for *B* is achieved over the complete energy range. In an earlier run of this experiment, *B* = 0.967(12) with a statistical uncertainty of ±0.006 was obtained [13]. If the electron and the proton are detected in opposite hemispheres, the direction of the neutrino is not so well defined and the asymmetry depends strongly on the electron energy. Furthermore, the result depends on a precise knowledge of the *β*-asymmetry parameter *A*. The highest sensitivity to *B* is in the low energy part of the spectrum where the electron spectroscopy is more difficult because of background and threshold effects. Considering these systematic effects, an evaluation of the asymmetry of Eq. (3.2) gives *B* = 0.91(6) [13].

## 5. Proton-Asymmetry *C*

The proton is measured in coincidence with a decay electron. With the proton count rates $N_{i}^{↑}\;(E_{e})\;$ and $N_{i}^{↓}\;(E_{e})\;$ as a function of the electron energy *E*_e_ in the two detectors (*i* = 1,2) for neutron spin up and down respectively, we define

$$\rho_{i}^{↑/↓}=\int N_{i}^{↑/↓}(E_{e})dE_{e}.$$ (4.1)

The quantity $\rho_{i}^{↑/↓}$ does not depend on *E*_e_. The experimental asymmetry $C_{i_{\exp}}$ is

$$C_{i_{\exp}}=\frac{\rho_{i}^{↑}-\rho_{i}^{↓}}{\rho_{i}^{↑}+\rho_{i}^{↓}}.$$ (4.2)

The proton-asymmetry *C* is expressed within the Standard Model with the parameters *A* and *B*

$$C_{i_{\exp}}=-Px_{1}(A+B).$$ (4.3)

and is thus sensitive to *λ* [14]

$$C_{i_{\exp}}=Px_{1}\frac{4\lambda }{1+3\lambda^{2}}.$$ (4.4)

*x*_1_ = 0.275 is a kinematical factor. In our recent measurement of the neutrino-asymmetry *B*, we automatically get $C_{i_{\exp}}$ = −0.238(11) [13] from a measurement of electrons and protons in coincidence, which is the first determination to date. A measurement of *C* has the potential to check results for *V_ud_* from *A* or *a* measurements or results for *B* with a precise *A* value.

## 5. Summary

This article has reported a new method for measuring the asymmetry coefficient *B*, which has allowed a value for the coefficient *C* to be obtained for the first time. A large improvement of the polarization uncertainties has now allowed a remeasurement of coefficient *A* with a reduction in the main correction from 1.1 % to less than 0.25 %, with an uncertainty of 0.1 %. Using the same quality of polarization, measurements of *B* and *C* are in progress.

## Figures and Tables

**Fig. 3:**
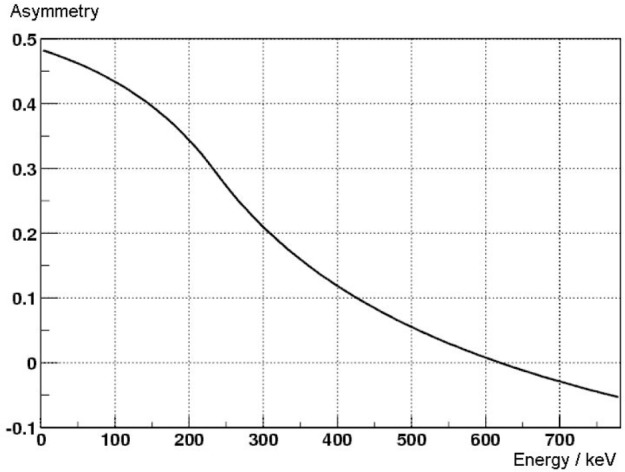
Expected proton-asymmetry $-{\hat{C}}_{i_{\exp}}=-\frac{N_{i}^{↑}-N_{i}^{↓}}{N_{i}^{↑}+N_{i}^{↓}}$ when measured in coincidence with decay electron.
